# Supplementary material for: Spx inhibits expression of the SwrA•DegU master flagellar activator in Bacillus subtilis
Source: J Bacteriol. 2025 Dec 5;208(1):e00415-25. doi: 10.1128/jb.00415-25 (PMC12826050; doi:10.1128/jb.00415-25)
Supplement: Supplemental tables and figures — Tables S1 to S3, and Figures S1 and S2. [file jb.00415-25-s0001.docx]

**TABLE S1: Plasmids**

| **Plasmid** | **Genotype** |
| --- | --- |
| pAEJ5  pAEJ9  pDG268  pDG1515  pDP538  pKM86  pMiniMAD2  pSG79  pSO18 | *amyE*::P*_degS_*-*lacZ cat amp*  *amyE*::P*_degU_*-*lacZ cat amp*  *amyE*::*lacZ* *cat amp*  *tet amp*  *ycgO*::P*_clpX_*-*clpX tet amp*  *ycgO*::*tet* *amp*  *mls ori^TsBs^ amp*  P*_fla/che_Ω*P*_hyspank_-fla/che* operon *kan*  Δ*clpX mls ori^TsBs^ amp* |

**TABLE S2: Primers**

| **Primer** | **Sequence** |
| --- | --- |
| 3250  3251  6333  6334  6335  6336  6589  6590  6591  7410  7411  7657  7658  7838  7839  7840  7841  7944  7945 | ACGACTCACTATAGGGCGAATTG  CTCACTAAAGGGAACAAAAGCTGG  attatttatcccaattagttggccttgaagcagg  tttaataagaggcgaggaggttgtgtttttccacagaacgagca  cgttctgtggaaaaacacaacctcctcgcctcttattaaaagac  aggagggtaccaaaaacctccaaacagcggga  AGGAGGAATTCAGCCCATATTGCTCGAGG  AGGAGGGATCCAAGGCTGATGACTCAGCCT  TTAAGAATAAAATAGATGTTCATCC  AGGAGCTCGAGCCTTGAAGTATCAGATGAGGAAG  ctcctgctagcgaataaacgtttttccggaagag  AGGAGGAATTCACCCTCCTGCTAAGCATAAAAGACTG  CTCCTGGATCCAATTTCCCTCCGTCACGGCGTTGTC  GGCTGCTGCGCGGCTTGTGTTGGAAG  CAATTCGCCCTATAGTGAGTCGTCTACATCTAAATGCAAAACCATCGTCGG  CCAGCTTTTGTTCCCTTTAGTGAGCCGGAAAGCGTCAGGGAGGGGCTGAC  GTCGACTACCGCATAAATTTCAGTGCGG  AGGAGGAATTCGGAATGCCCTTTACGAAGTGAG  CTCCTAAGCTTATTGTTCTATATACTCTAGCATTCGG |

**Table S3: Raw data from β-galactosidase assays.^1^**

|  | **wild**  **type** | ***clpX*** | ***clpX***  **(*clpX*)** | ***clpX***  ***lonA*** | ***clpX spx*** | ***clpX***  ***lonA spx*** |
| --- | --- | --- | --- | --- | --- | --- |
| +*amyE*::  P*_hag_*-*lacZ* | 464 ± 75.7 (DK5457) | 172 ± 22.9 (DB20) | 440 ± 9.8 (DB26) | 375 ± 41.7 (DB28) | 333 ± 14.6 (DB24) | 508 ± 43.6 (DB54) |
| +*amyE*::  P*_fla/che_*-*lacZ* | 459 ± 20.7 (DK2399) | 169 ± 3.2 (DB67) | 417 ± 30.4 (DB68) | 236 ± 57.9 (DB70) | 454 ± 33.8 (DB69) | 573 ± 25.8 (DB71) |
| +*amyE*::  P*_degS_*-*lacZ* | 8 ± 0.4 (DK9529) | 11 ± 0.9 (DB1527) | 5 ± 0.3 (DB1529) | 10 ± 1.0 (DB1530) | 4 ± 1.2 (DB1528) | 5 ± 0.6 (DB1531) |
| +*amyE*::  P*_degU_*-*lacZ* | 5 ± 1.0 (DB559) | 1 ± 0.1 (DB807) | 4 ± 0.8 (DB811) | 2 ± 0.3 (DB813) | 8 ± 0.6 (DB809) | 14 ± 3.6 (DB848) |
| +*amyE*::  P*_swrA_*-*lacZ* | 37 ± 2.5 (DK6613) | 10 ± 0.6 (DB806) | 38 ± 2.3 (DB810) | 18 ± 2.8 (DB812) | 31 ± 1.5 (DB808) | 50 ± 2.9 (DB847) |

**SUPPLEMENTAL FIGURE LEGENDS**

**Figure S1. Mutation of the anti-sigma factor FlgM does not restore swarming in cells mutated for ClpX.** Quantitative swarm expansion assays for the strains indicated in each panel. Genes in italics are mutated, genes in parenthesis are complementation constructs expressed under the gene’s native promoter and inserted at an ectopic site in the chromosome, and a “+” indicates that the gene was induced with 1 mM IPTG. Each data point is the average of three technical replicates. (A) The *clpX* mutant phenotype was not rescued when the gene for the anti-sigma factor, *flgM*, was also mutated. The following strains were used to generate the data in this panel: WT (DK1042), *clpX* (DK6563), *clpX* *flgM* (DB3069). **(B)** The partially swarming *clpX* *degU*^+^ strain was improved when the gene for the anti-sigma factor, *flgM*, was also mutated. The following strains were used to generate the data in this panel: *degU*^+^ (DB644, 1 mM IPTG added), *clpX* *degU*^+^ *flgM* (DB3070, 1 mM IPTG added), *clpX* *degU*^+^ (DB470, 1 mM IPTG added), *clpX* (DB470, 0 mM IPTG added).

**Figure S2. Spontaneous suppressor mutations restore swarming in cells mutated for ClpX.** Quantitative swarm expansion assays for the strains indicated in each panel. “*sox*” or “*asx*” indicate suppressor of *clpX* isolated from separate experiments. Genes in italics are mutated. Each data point is the average of three technical replicates. (A) The *clpX* mutant phenotype was rescued by a single nucleotide polymorphism in P*_fla/che_* (*sox16*). The following strains were used to generate the data in this panel: WT (DK1042), *sox16* (DK9447), *clpX* (DK6563). (B) The *clpX* mutant phenotype was rescued by a 25-nucleotide deletion including the terminator for *codY* (*sox15*). The following strains were used to generate the data in this panel: *sox15* (DK9750), WT (DK1042), *clpX* (DK6563). (C) The *clpX* mutant phenotype was rescued by mutations in *lonA*: *sox9* and *sox12* were slipped-strand mispairing mutations and *sox17* was an insertion of 23 nucleotides. The following strains were used to generate the data in this panel: WT (DK1042), *sox9* (DK9748), *sox17* (DK9751), *sox12* (DK9749), *clpX* (DK6563). (D) The *clpX* mutant phenotype was rescued by deletions in the *spx* coding region: *sox2* was a deletion of coding nucleotides, and *sox18* and *sox19* were deletions including the promoter and coding nucleotides. The following strains were used to generate the data in this panel: WT (DK1042), *sox2* (DK9386), *sox19* (DK9391), *sox18* (DK9390), *clpX* (DK6563). (E) The *clpX* mutant phenotype was rescued by different large deletions of multiple genes that included *spx* (*sox1* and *sox6*). The following strains were used to generate the data in this panel: WT (DK1042), *sox6* (DK9746), *sox1* (DK9744), *clpX* (DK6563). (F) The *clpX* mutant phenotype was rescued by a loss-of-function SNP in P*_spx_* (*sox5*, *sox10*, *sox11*, and *sox13*). Only *sox5* is shown. The following strains were used to generate the data in this panel: WT (DK1042), *sox5* (DK9393), *clpX* (DK6563). (G) The *clpX* mutant phenotype was rescued by missense mutations in the *spx* coding region: *sox14* = *spx*^R119H^, *sox3* = *spx*^F113C^, *sox20* = *spx*^V2F^, *sox7* = *spx*^Y5C^. The following strains were used to generate the data in these panels: WT (DK1042), *sox14* (DK9389), *sox3* (DK9387), *sox20* (DK9392), *sox7* (DK9388), *clpX* (DK6563). (H) The *clpX* mutant phenotype was rescued by more missense mutations in the *spx* coding region: *asx27* = *spx*^T49I^, *asx17* = *spx*^P93L^, *asx6* = *spx*^Y80H^. Suppressors are shown in two separate panels for simplicity. The following strains were used to generate the data in these panels: WT (DK1042), *asx27* (DK9865), *asx17* (DK9843), *asx6* (DK9753), *clpX* (DK6563). (I) The *clpX* mutant phenotype was rescued by an inserted glycine at amino acid position 53 in *spx* (*asx4*). The following strains were used to generate the data in this panel: WT (DK1042), *asx4* (DK9842), *clpX* (DK6563). See Table 1 for more detailed information on suppressor mutations.
